# (2*E*,4*E*)-1-(2-Hy­droxy­phen­yl)-5-phenyl­penta-2,4-dien-1-one

**DOI:** 10.1107/S160053681103025X

**Published:** 2011-08-02

**Authors:** W. A. Silva, C. C. Gatto, G. R. Oliveira

**Affiliations:** a Instituto de Química – IQ, Universidade de Brasília – UnB, Campus Universitário Darcy Ribeiro, Caixa postal 04478, Asa Norte Brasília DF, CEP 70904-970, Brazil; b Instituto de Química – IQ, Universidade Federal de Goiás, CP 131, Campus Samambaia, Goiânia GO, CEP 74001-970, Brazil

## Abstract

In the structure of the title chalcone, C_17_H_14_O_2_, derived from cinnamaldehyde, the olefine group has a *trans* configuration. The mol­ecular conformation is stabilized by an intra­molecular O—H⋯O hydrogen-bond inter­action with graph-set motif *S*(6).

## Related literature

For the preparation, see: Lawrence *et al.* (2001 ▶). For related structures, see: Patil *et al.* (2007 ▶); Zhao *et al.* (2007 ▶). For standard bond lengths, see: Allen (2002 ▶). For hydrogen-bond motifs, see: Bernstein *et al.* (1995 ▶). For related activity and structures, see: Dyrager *et al.* (2011 ▶); Jasinski *et al.* (2009 ▶); Ruan *et al.* (2011 ▶); Vencato *et al.* (2006 ▶). 
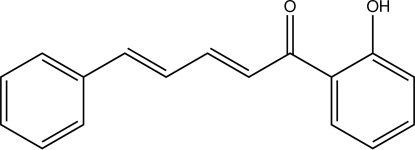


## Experimental

### 

#### Crystal data

C_17_H_14_O_2_
*M* *_r_* = 250.28
Orthorhombic, 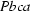

*a* = 10.9068 (3) Å
*b* = 7.9851 (2) Å
*c* = 30.2131 (7) Å
*V* = 2631.32 (12) Å^3^
*Z* = 8
Mo *K*α radiation
μ = 0.08 mm^−1^
*T* = 296 K
0.39 × 0.17 × 0.09 mm

#### Data collection

Bruker X8 SMART APEXII diffractometer
Absorption correction: multi-scan (*SADABS*; Bruker, 2008 ▶) *T*_min_ = 0.92, *T*_max_ = 0.99
18214 measured reflections
2691 independent reflections
1479 reflections with *I* > 2σ(*I*)
*R*_int_ = 0.059

#### Refinement

*R*[*F* ^2^ > 2σ(*F* ^2^)] = 0.050
*wR*(*F* ^2^) = 0.134
*S* = 1.01
2691 reflections
174 parameters
H-atom parameters constrained
Δρ_max_ = 0.19 e Å^−3^
Δρ_min_ = −0.15 e Å^−3^

### 

Data collection: *APEX2* (Bruker, 2009 ▶); cell refinement: *SAINT* (Bruker, 2009 ▶); data reduction: *SAINT*; program(s) used to solve structure: *SHELXS97* (Sheldrick, 2008 ▶); program(s) used to refine structure: *SHELXL97* (Sheldrick, 2008 ▶); molecular graphics: *ORTEP-3 for Windows* (Farrugia, 1997 ▶); software used to prepare material for publication: *publCIF* (Westrip, 2010) ▶.

## Supplementary Material

Crystal structure: contains datablock(s) global, I. DOI: 10.1107/S160053681103025X/bx2361sup1.cif

Structure factors: contains datablock(s) I. DOI: 10.1107/S160053681103025X/bx2361Isup2.hkl

Supplementary material file. DOI: 10.1107/S160053681103025X/bx2361Isup3.cml

Additional supplementary materials:  crystallographic information; 3D view; checkCIF report

## Figures and Tables

**Table 1:** Hydrogen-bond geometry (Å, °)

*D*—H⋯*A*	*D*—H	H⋯*A*	*D*⋯*A*	*D*—H⋯*A*
O1—H1⋯O2	0.82	1.81	2.5317 (19)	146

**Fig. 1.**
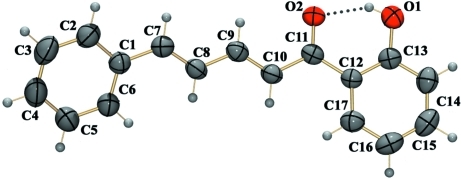
The molecular structure of showing the atomic labelling scheme. The anisotropic displacement parameters are at the 50% level. The dashed line indicates an intramolecular hydrogen bond, O1—H1···O2.

## supplementary crystallographic information

### Comment

A large number of chalcones showed significant activity against many diseases. Some results from our laboratory showed that the synthesis of analogues of chalcones derived from cinnamaldehyde was often accompanied by increased in vitro bioactivity. However, the synthesis and analysis cytotoxic of these analogs of chalcones appears to be a largely unexplored field. With that, needs to make an accurate study of this new class of compounds in order to improve their structure-activity relationships.

The configuration of the olefinic group is trans, this was caractherized for 1HNMR where was showed the coupling J trans is around 15 Hz [C8—C9—C10—C11]. The presence of α-β-unsaturated ketone is indicated by the short O2–C11 and C9–C10 bond lengths of 1.245 (3) and 1.334 (3) Å, respectively, and the O2–C11–C10 and C9–C10–C11 bond angles of 118.8 (2)° and 121.8 (2)°, respectively. The bond distances are of normal values and are comparable with those found in related structures [Zhao *et al.* (2007); Patil *et al.* (2007)]. The molecular conformation is stabilized by one intramolecular O—H···O hydrogen-bond interaction with set graph motif S(6), (Bernstein *et al.*, 1995) [O1···O2 2.5285 (19) Å, O1–H1···O2 146.4°] (Fig. 2).

### Experimental

Compound was obtained by the aldol condensation of cinnamaldehyde, and 2-hidroxyacetophenone, using a method described previously [Lawrence *et al.* 2001]. Crystals were obtained from an EtOH solution (m.p. 428 K). Yield = 80% 1H NMR: δ 6.92 (ddd, J = 7.8, 7.6 and 1.1 Hz, 1 H, 16-H), 7.01 (dd, J = 8.1 and 1.1 Hz, 1 H, 14- H), 7.05–7.07 (m, 2 H, 8, 7-H), 7.22 (d, J = 14.7 Hz, 1 H, 10-H), 7.34–7.42 (m, 3 H, 3,4,5-H), 7.49 (ddd, J = 8.1, 7.6 and 1.4 Hz, 1 H, 15-H), 7.52 (dd, J = 7.8 and 1.7 Hz, 2 H, 2,6-H), 7.67–7.76 (m, 1 H, 9-H), 7.85 (dd, J = 7.8 and 1.4 Hz, 1 H, 17-H), 12.88 (s, 1 H, OH). 13 C NMR: δ = 118.6 (C-14), 118.8 (C-16), 120.0 (C-12), 123.5 (C-10), 126.7 (C-8), 127.4 (C-2,6), 128.9 (C-3,5), 129.4 (C-4), 129.5 (C-17), 135.9 (C-1), 136.2 (C-15), 142.9 (C-7), 145.4 (C-9), 163.5 (C-13), 194.0 (C11).

### Refinement

All H atoms were positioned geometrically and allowed to ride on their parent atoms, with d(C—H) = 0.93 Å and *U*_iso_ = 1.2Ueq(C).

### Crystal data

**Table:**

C_17_H_14_O_2_	*D*_x_ = 1.264 Mg m^−^^3^
*M**_r_* = 250.28	Melting point: 428 K
Orthorhombic, *P**b**c**a*	Mo *K*α radiation, λ = 0.71073 Å
*a* = 10.9068 (3) Å	Cell parameters from 1573 reflections
*b* = 7.9851 (2) Å	θ = 2.7–19.5°
*c* = 30.2131 (7) Å	µ = 0.08 mm^−^^1^
*V* = 2631.32 (12) Å^3^	*T* = 296 K
*Z* = 8	Block, yellow
*F*(000) = 1056	0.39 × 0.17 × 0.09 mm

### Data collection

**Table:**

Bruker X8 SMART APEXII diffractometer	2691 independent reflections
graphite	1479 reflections with *I* > 2σ(*I*)
Detector resolution: 8.3333 pixels mm^-1^	*R*_int_ = 0.059
φ and ω scans	θ_max_ = 26.4°, θ_min_ = 1.4°
Absorption correction: multi-scan (*SADABS*; Bruker, 2008)	*h* = −13→11
*T*_min_ = 0.92, *T*_max_ = 0.99	*k* = −9→9
18214 measured reflections	*l* = −36→37

### Refinement

**Table:**

Refinement on *F*^2^	Secondary atom site location: difference Fourier map
Least-squares matrix: full	Hydrogen site location: inferred from neighbouring sites
*R*[*F*^2^ > 2σ(*F*^2^)] = 0.050	H-atom parameters constrained
*wR*(*F*^2^) = 0.134	*w* = 1/[σ^2^(*F*_o_^2^) + (0.0634*P*)^2^] where *P* = (*F*_o_^2^ + 2*F*_c_^2^)/3
*S* = 1.01	(Δ/σ)_max_ = 0.001
2691 reflections	Δρ_max_ = 0.19 e Å^−^^3^
174 parameters	Δρ_min_ = −0.15 e Å^−^^3^
0 restraints	Extinction correction: *SHELXL*
Primary atom site location: structure-invariant direct methods	Extinction coefficient: 0.0109 (11)

### Special details

**Table:**

Geometry. All e.s.d.'s (except the e.s.d. in the dihedral angle between two l.s. planes) are estimated using the full covariance matrix. The cell e.s.d.'s are taken into account individually in the estimation of e.s.d.'s in distances, angles and torsion angles; correlations between e.s.d.'s in cell parameters are only used when they are defined by crystal symmetry. An approximate (isotropic) treatment of cell e.s.d.'s is used for estimating e.s.d.'s involving l.s. planes.
Refinement. Refinement of *F*^2^ against ALL reflections. The weighted *R*-factor *wR* and goodness of fit *S* are based on *F*^2^, conventional *R*-factors *R* are based on *F*, with *F* set to zero for negative *F*^2^. The threshold expression of *F*^2^ > σ(*F*^2^) is used only for calculating *R*-factors(gt) *etc*. and is not relevant to the choice of reflections for refinement. *R*-factors based on *F*^2^ are statistically about twice as large as those based on *F*, and *R*- factors based on ALL data will be even larger.

### Fractional atomic coordinates and isotropic or equivalent isotropic displacement parameters (Å^2^)

**Table:**

	*x*	*y*	*z*	*U*_iso_*/*U*_eq_	
O2	0.09900 (13)	0.62840 (18)	1.00369 (4)	0.0625 (4)	
O1	0.07689 (13)	0.4619 (2)	1.07455 (5)	0.0652 (5)	
H1	0.0543	0.5068	1.0515	0.098*	
C12	0.27179 (18)	0.5570 (2)	1.04643 (6)	0.0443 (5)	
C13	0.19982 (19)	0.4740 (2)	1.07803 (6)	0.0488 (5)	
C11	0.21235 (18)	0.6346 (2)	1.00794 (6)	0.0472 (5)	
C9	0.23958 (18)	0.7440 (2)	0.93309 (6)	0.0511 (5)	
H9	0.1581	0.7152	0.9281	0.061*	
C1	0.33154 (19)	0.8908 (3)	0.81740 (6)	0.0532 (5)	
C10	0.28508 (18)	0.7155 (2)	0.97335 (6)	0.0515 (5)	
H10	0.3651	0.748	0.9796	0.062*	
C17	0.39835 (19)	0.5612 (3)	1.05313 (7)	0.0567 (6)	
H17	0.4476	0.6164	1.0327	0.068*	
C8	0.30670 (19)	0.8155 (2)	0.89703 (6)	0.0535 (5)	
H8	0.3844	0.8579	0.903	0.064*	
C7	0.26596 (19)	0.8255 (2)	0.85581 (7)	0.0554 (6)	
H7	0.1868	0.7866	0.8508	0.066*	
C6	0.4439 (2)	0.9703 (3)	0.82097 (7)	0.0620 (6)	
H6	0.4796	0.9844	0.8487	0.074*	
C5	0.5037 (2)	1.0290 (3)	0.78378 (8)	0.0762 (7)	
H5	0.5794	1.0815	0.7866	0.091*	
C15	0.3789 (2)	0.4036 (3)	1.11921 (7)	0.0686 (6)	
H15	0.4151	0.3511	1.1434	0.082*	
C16	0.4517 (2)	0.4863 (3)	1.08888 (7)	0.0671 (6)	
H16	0.5362	0.491	1.0928	0.081*	
C14	0.2542 (2)	0.3976 (3)	1.11417 (7)	0.0627 (6)	
H14	0.2062	0.3423	1.135	0.075*	
C2	0.2808 (2)	0.8734 (3)	0.77533 (7)	0.0663 (7)	
H2	0.2052	0.8209	0.7721	0.08*	
C3	0.3414 (3)	0.9331 (3)	0.73832 (7)	0.0791 (8)	
H3	0.3065	0.9205	0.7104	0.095*	
C4	0.4522 (3)	1.0105 (4)	0.74271 (8)	0.0822 (8)	
H4	0.4929	1.0507	0.7178	0.099*	

### Atomic displacement parameters (Å^2^)

**Table:**

	*U*^11^	*U*^22^	*U*^33^	*U*^12^	*U*^13^	*U*^23^
O2	0.0432 (9)	0.0774 (10)	0.0669 (10)	−0.0019 (7)	−0.0019 (7)	0.0144 (7)
O1	0.0552 (10)	0.0791 (11)	0.0615 (10)	−0.0017 (8)	0.0075 (7)	0.0119 (8)
C12	0.0438 (13)	0.0442 (11)	0.0450 (11)	0.0031 (9)	0.0010 (9)	−0.0063 (9)
C13	0.0491 (13)	0.0487 (11)	0.0485 (11)	0.0033 (10)	0.0014 (10)	−0.0078 (9)
C11	0.0418 (12)	0.0468 (11)	0.0529 (12)	−0.0010 (9)	0.0037 (10)	−0.0051 (9)
C9	0.0454 (13)	0.0494 (12)	0.0585 (13)	0.0008 (10)	0.0042 (10)	−0.0005 (10)
C1	0.0542 (15)	0.0540 (12)	0.0515 (13)	0.0060 (11)	0.0009 (10)	0.0018 (9)
C10	0.0447 (13)	0.0557 (12)	0.0540 (12)	−0.0048 (10)	−0.0008 (10)	−0.0004 (10)
C17	0.0505 (14)	0.0619 (14)	0.0577 (13)	0.0023 (10)	−0.0012 (11)	−0.0059 (10)
C8	0.0468 (12)	0.0561 (12)	0.0577 (13)	−0.0039 (10)	0.0033 (10)	0.0043 (10)
C7	0.0499 (13)	0.0580 (13)	0.0583 (13)	−0.0028 (10)	−0.0025 (11)	0.0024 (10)
C6	0.0575 (15)	0.0729 (15)	0.0555 (14)	−0.0001 (12)	0.0010 (11)	0.0080 (11)
C5	0.0616 (16)	0.0920 (18)	0.0748 (17)	0.0004 (13)	0.0064 (13)	0.0201 (14)
C15	0.0813 (19)	0.0689 (14)	0.0555 (14)	0.0150 (13)	−0.0163 (13)	−0.0059 (12)
C16	0.0564 (15)	0.0764 (16)	0.0684 (16)	0.0061 (12)	−0.0148 (12)	−0.0087 (13)
C14	0.0792 (17)	0.0614 (14)	0.0476 (12)	0.0033 (12)	0.0012 (12)	−0.0001 (10)
C2	0.0660 (16)	0.0763 (16)	0.0566 (14)	0.0051 (12)	−0.0074 (12)	0.0013 (11)
C3	0.086 (2)	0.101 (2)	0.0504 (14)	0.0173 (16)	−0.0046 (13)	0.0067 (13)
C4	0.0790 (19)	0.107 (2)	0.0610 (17)	0.0177 (16)	0.0127 (14)	0.0238 (14)

### Geometric parameters (Å, °)

**Table:**

O2—C11	1.244 (2)	C8—C7	1.324 (3)
O1—C13	1.348 (2)	C8—H8	0.93
O1—H1	0.82	C7—H7	0.93
C12—C17	1.395 (3)	C6—C5	1.381 (3)
C12—C13	1.402 (3)	C6—H6	0.93
C12—C11	1.469 (3)	C5—C4	1.370 (3)
C13—C14	1.384 (3)	C5—H5	0.93
C11—C10	1.463 (3)	C15—C14	1.369 (3)
C9—C10	1.333 (2)	C15—C16	1.381 (3)
C9—C8	1.431 (3)	C15—H15	0.93
C9—H9	0.93	C16—H16	0.93
C1—C6	1.385 (3)	C14—H14	0.93
C1—C2	1.393 (3)	C2—C3	1.383 (3)
C1—C7	1.460 (3)	C2—H2	0.93
C10—H10	0.93	C3—C4	1.364 (3)
C17—C16	1.365 (3)	C3—H3	0.93
C17—H17	0.93	C4—H4	0.93
			
C13—O1—H1	109.5	C8—C7—H7	116.4
C17—C12—C13	117.80 (18)	C1—C7—H7	116.4
C17—C12—C11	122.77 (18)	C5—C6—C1	120.6 (2)
C13—C12—C11	119.44 (18)	C5—C6—H6	119.7
O1—C13—C14	117.13 (19)	C1—C6—H6	119.7
O1—C13—C12	122.51 (18)	C4—C5—C6	120.5 (2)
C14—C13—C12	120.4 (2)	C4—C5—H5	119.7
O2—C11—C10	118.87 (17)	C6—C5—H5	119.7
O2—C11—C12	120.23 (17)	C14—C15—C16	120.9 (2)
C10—C11—C12	120.87 (17)	C14—C15—H15	119.5
C10—C9—C8	124.9 (2)	C16—C15—H15	119.5
C10—C9—H9	117.6	C17—C16—C15	119.3 (2)
C8—C9—H9	117.6	C17—C16—H16	120.3
C6—C1—C2	117.93 (19)	C15—C16—H16	120.3
C6—C1—C7	122.38 (18)	C15—C14—C13	119.9 (2)
C2—C1—C7	119.7 (2)	C15—C14—H14	120.1
C9—C10—C11	121.70 (19)	C13—C14—H14	120.1
C9—C10—H10	119.2	C3—C2—C1	120.9 (2)
C11—C10—H10	119.2	C3—C2—H2	119.5
C16—C17—C12	121.7 (2)	C1—C2—H2	119.5
C16—C17—H17	119.1	C4—C3—C2	120.0 (2)
C12—C17—H17	119.1	C4—C3—H3	120.0
C7—C8—C9	124.6 (2)	C2—C3—H3	120.0
C7—C8—H8	117.7	C3—C4—C5	120.0 (2)
C9—C8—H8	117.7	C3—C4—H4	120.0
C8—C7—C1	127.2 (2)	C5—C4—H4	120.0
			
C17—C12—C13—O1	−179.60 (17)	C6—C1—C7—C8	−7.7 (3)
C11—C12—C13—O1	0.2 (3)	C2—C1—C7—C8	172.1 (2)
C17—C12—C13—C14	−0.8 (3)	C2—C1—C6—C5	−0.5 (3)
C11—C12—C13—C14	178.95 (17)	C7—C1—C6—C5	179.4 (2)
C17—C12—C11—O2	−179.47 (18)	C1—C6—C5—C4	0.5 (4)
C13—C12—C11—O2	0.8 (3)	C12—C17—C16—C15	0.3 (3)
C17—C12—C11—C10	2.5 (3)	C14—C15—C16—C17	−0.9 (3)
C13—C12—C11—C10	−177.20 (17)	C16—C15—C14—C13	0.6 (3)
C8—C9—C10—C11	−177.06 (18)	O1—C13—C14—C15	179.10 (18)
O2—C11—C10—C9	−17.4 (3)	C12—C13—C14—C15	0.2 (3)
C12—C11—C10—C9	160.63 (18)	C6—C1—C2—C3	0.3 (3)
C13—C12—C17—C16	0.5 (3)	C7—C1—C2—C3	−179.6 (2)
C11—C12—C17—C16	−179.20 (18)	C1—C2—C3—C4	−0.1 (4)
C10—C9—C8—C7	171.7 (2)	C2—C3—C4—C5	0.0 (4)
C9—C8—C7—C1	−177.45 (19)	C6—C5—C4—C3	−0.2 (4)

### Hydrogen-bond geometry (Å, °)

**Table:**

*D*—H···*A*	*D*—H	H···*A*	*D*···*A*	*D*—H···*A*
O1—H1···O2	0.82	1.81	2.5317 (19)	146.
